# Synthesis and reactivity of a trigonal porous nanographene on a gold surface†
†Electronic supplementary information (ESI) available. See DOI: 10.1039/c9sc03404h


**DOI:** 10.1039/c9sc03404h

**Published:** 2019-09-13

**Authors:** Rafal Zuzak, Iago Pozo, Mads Engelund, Aran Garcia-Lekue, Manuel Vilas-Varela, José M. Alonso, Marek Szymonski, Enrique Guitián, Dolores Pérez, Szymon Godlewski, Diego Peña

**Affiliations:** a Centre for Nanometer-Scale Science and Advanced Materials, NANOSAM , Faculty of Physics , Astronomy and Applied Computer Science , Jagiellonian University , Łojasiewicza 11 , PL 30-348 Kraków , Poland . Email: szymon.godlewski@uj.edu.pl; b Centro de Investigación en Química Biolóxica e Materiais Moleculares (CiQUS) , Departamento de Química Orgánica , Universidade de Santiago de Compostela , 15782-Santiago de Compostela , Spain . Email: diego.pena@usc.es; c Espeem S.A.R.L. , L-4365 Esch-sur-Alzette , Luxembourg; d Donostia International Physics Center, DIPC , Paseo Manuel de Lardizabal 4 , E-20018 Donostia-San Sebastián , Spain; e IKERBASQUE , Basque Foundation for Science , E-48013 Bilbao , Spain

## Abstract

The synthesis of porous nanographenes is a challenging task for solution chemistry, and thus, on-surface synthesis provides an alternative approach. Here, we report the synthesis of a triporous nanographene with 102 sp^2^ carbon atoms by combining solution and surface chemistry. The carbon skeleton was obtained by Pd-catalyzed cyclotrimerization of arynes in solution, while planarization of the molecule was achieved through two hierarchically organized on-surface cyclodehydrogenation reactions, intra- and inter-blade. Remarkably, the three non-planar [14]annulene pores of this nanographene further evolved at higher temperatures showing interesting intra-porous on-surface reactivity.

## Abstract 2

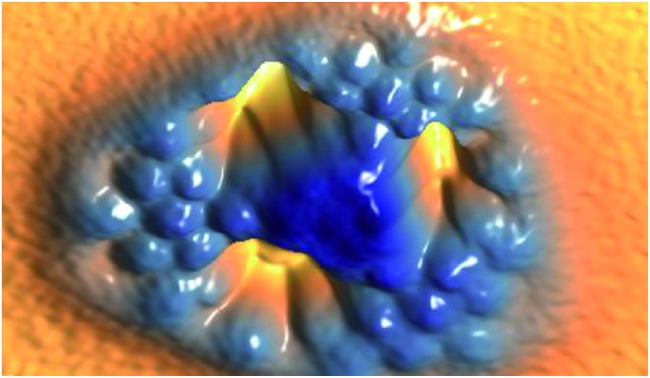
Synthesis of a triporous nanographene with 102 sp^2^ carbon atoms by combining solution and surface chemistry.

## Introduction

Recently, nanographenes have attracted much attention in materials sciences due to their unique optoelectronics properties.^1^ This interest has motivated the search for efficient methods to prepare well-defined nanographenes by chemical synthesis. In particular, nanographenes with embedded pores in their structure are specially interesting materials, since the pores can significantly influence the electronic properties of these graphene derivatives. In addition, porous graphenes can be used in a large variety of applications, including sequencing, gas separation and sieving. However, the available synthetic methodologies to prepare well-defined porous nanographenes are quite limited.^2^–^4^ In this respect, an interesting approach is the combination of solution and on-surface chemistry.^5^,^6^ For example, this strategy has recently allowed the bottom–up preparation of a multifunctional nanoporous graphene.^7^

**Fig. 1:**
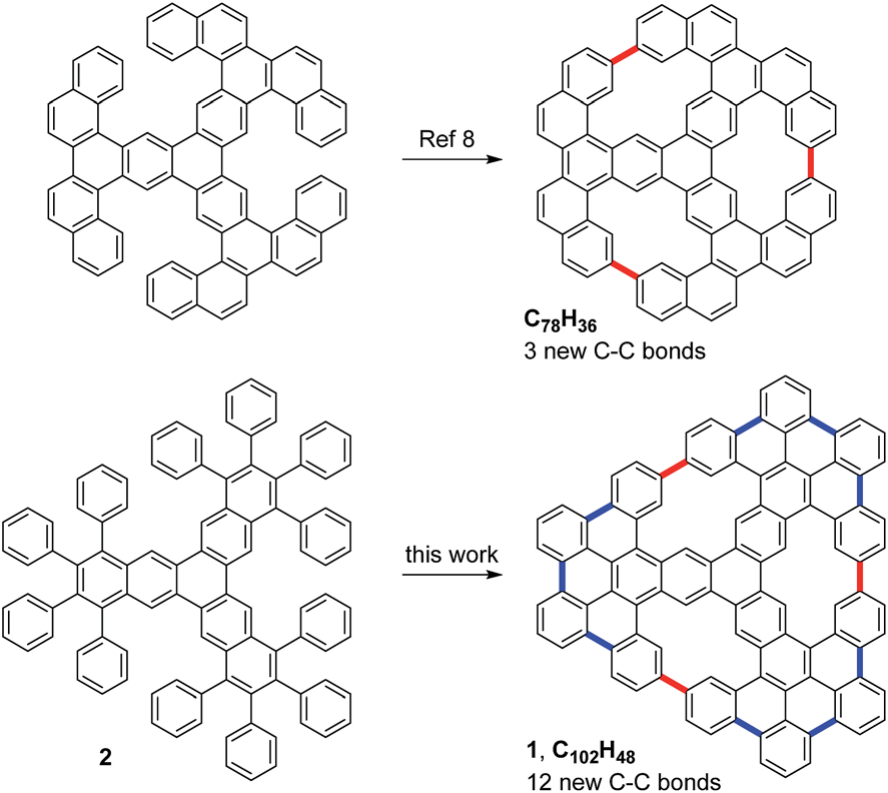
Structures of two porous nanographenes with three embedded [14]annulene pores.

Very recently, Müllen, Fasel, Feng and co-workers reported on the preparation of a porous nanographene with 78 sp^2^ carbon atoms by the on-surface cyclodehydrogenation of a starphene derivative through the formation of three C–C bonds (Fig. 1).^8^ Concurrently, we envisioned the synthesis of the related porous nanographene **1**, with 102 sp^2^ carbon atoms and three non-planar [14]annulene pores within its structure. We plan to obtain this nanographene starting with dodecaphenyl[7]starphene **2**, which could be easily obtained by means of aryne chemistry in solution.^9^ Then, sequential on-surface cyclodehydrogenations through the formation of nine intra-blade C–C bonds (in blue) and three inter-blade C–C bonds (in red) would afford trigonal porous nanographene **1**.

## Results and discussion

**Fig. 2:**
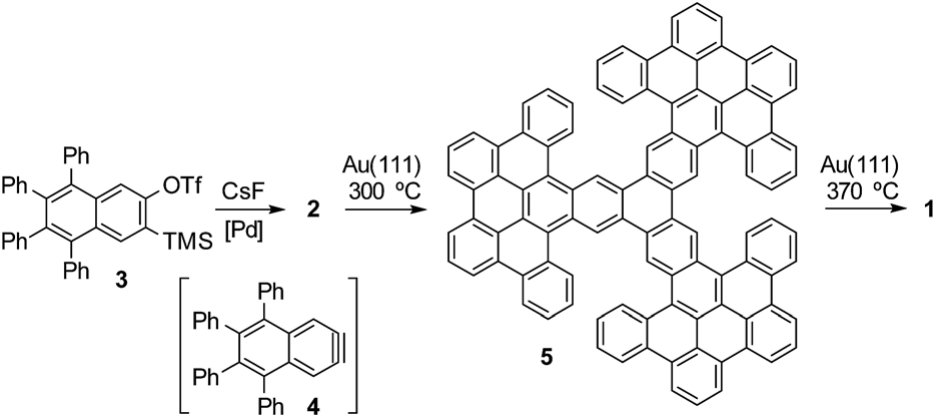
Synthetic route to obtain porous nanographene **1**.

We started the synthetic route by preparing triflate **3** (Fig. 2), which should be an adequate precursor of aryne **4** (ESI† for details). According to our well-established methodology for the palladium-catalyzed cyclotrimerization of arynes,9*d* subsequent generation of aryne **4** by fluoride-induced decomposition of triflate **3** in the presence of Pd(PPh_3_)_4_ led to the isolation of dodecaphenyl[7]starphene **2** in 37% yield. Then, we attempted the preparation of porous nanographene **1** by DDQ-promoted cyclodehydrogenation of compound **2** in solution. Unfortunately, this approach was unsuccessful since we only detected the formation of nanographene **5** as the result of nine intra-blade cyclodehydrogenations. Based on our recent experience,^10^ we attempted the preparation of **1** by on-surface cyclodehydrogenation of **5** through inter-blade C–C bond formation. However, the deposition of compound **5** on a gold surface by sublimation from an effusion cell proved to be infeasible, most probably due to significant intermolecular interactions. Then, we decided to study sequential on-surface cyclodehydrogenations of trimer **2** as explained below.

After thermal deposition of precursors **2** on Au(111), we found that these molecules tend to gather into disordered π-stacked assemblies, whose structures are difficult to determine by STM. This is due to the high degree of conformational flexibility due to the rotation of the peripheral phenyl rings. This flexibility combined with the steric hindrance between neighboring phenyl rings leads to the molecules adopting a non-planar geometry. On a metal surface, which is known to catalyse cyclodehydrogenation reactions,^11^ intramolecular fusion between neighboring phenyl rings shall occur. Intuitively, at first we might envision ring closure between phenyls attached to the very same naphthalene moiety of the molecule core (intra-blade). This would transform trinaphthylene **2** into the polycyclic three-bladed propeller **5**. Each of the wide propeller blades would be then composed of 9 rings. It is instructive to note that the transformed molecule would retain the non-planar character due to the steric interactions between the outermost rings of neighboring propeller blades within the necking of the conjoined bay regions.

**Fig. 3:**
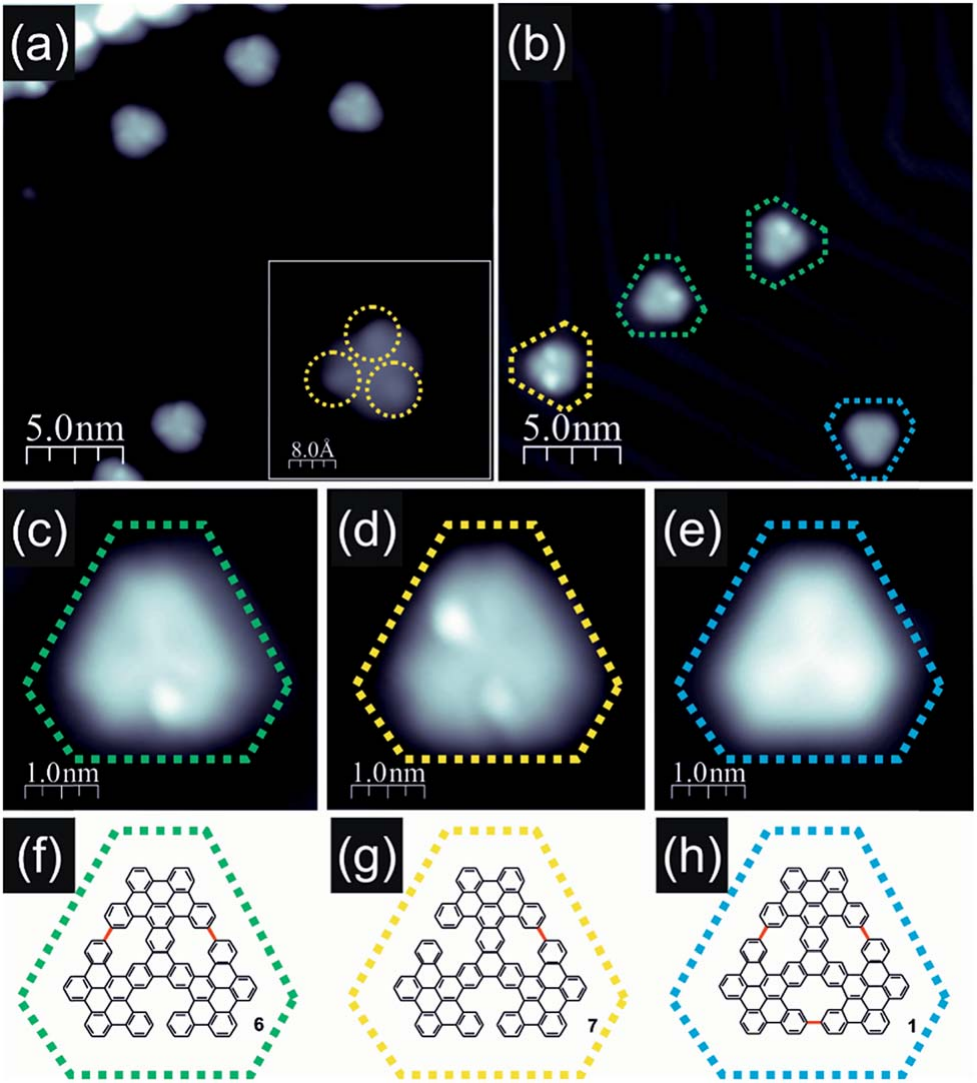
Products detected after thermal annealing of trinaphthalene **2** on Au(111); (a) filled state STM image of molecules **5** obtained after annealing of precursor **2** at 300 °C, which led to intra-blade cyclodehydrogenation and formation of intermediate **5**; the inset shows the STM topography of a single molecule **5** with three clearly discernible bright protrusions marked with dashed yellow circles; (b) filled state STM image of molecules obtained after precursor annealing at 370 °C; green, yellow and blue circles denote molecules with two, one and no bright protrusions in STM images, respectively; (c–e) high resolution filled state STM images of molecules **6**, **7** and **1** obtained after annealing at 370 °C drawn together with anticipated structural schemes (f–h); color coding identical to that in panel (b), new C–C bonds closing [14]annulene pores are highlighted in red within (f–h) schemes; imaging parameters: tunnelling current 100 pA, bias voltage –1.0 V.

Indeed, annealing at 300–340 °C induced considerable modification of STM images: the disordered assemblies were gone and we found the molecules as individual entities dispersed over the surface area. Closer inspection of the recorded STM images led to the conclusion that compound **5** was formed as the major reaction product (Fig. 3a). In fact, the individual molecules were recorded as triangular objects with rounded vertices, while their internal contrast was non-uniform. The STM images showed three bright lobes, each located within one of the longer molecule edges as clearly discernible in Fig. 3a. Simple comparison between the anticipated shape of compound **5** and the STM topographies suggested that the lobes appearing bright may correspond to the necking of the conjoined bay regions, where the steric hindrance led to out-of-plane dent of the neighboring propeller blades. The appearance of three bright lobes might be thus associated with the repulsive steric interaction between each pair of propeller blades and consequently also their bending. Minor differences among the recorded STM topographies of the molecules, expressed mainly in the details of the bright lobes' location and intensities, might be associated with differences in geometrical conformation. These differences arose from the opposite bending directions of neighboring propeller blades surrounding the conjoined bay region in **5**. Such a process shall occur within each pair of propeller blades giving rise to numerous stereoisomers with pairs of blades differently bent and consequently providing a variety of slightly different STM images.

Further transformation of compound **5** was achieved when the annealing temperature was slightly raised to the range of approximately 340–380 °C. The molecules were still distributed over the surface and their STM images retained a triangular shape; however, the internal contrast was different. We noticed that unlike in the case of lower temperature annealing, now we could not detect molecules exhibiting 3 bright lobes (compound **5**) as documented in Fig. 3b. Instead, the molecules could be classified into three co-existing groups depending on the number of bright lobes recorded for a single molecule. They are visualized in Fig. 3c–e. The first group (Fig. 3c) contained only one bright lobe, the second family exhibited two bright lobes (Fig. 3d) and the last third one gathered molecules which do not exhibit any bright protrusion (Fig. 3e). The latter group could be characterized by a three-fold symmetric appearance with a slightly brighter Y-shape in the centre indicating the successful generation of porous nanographene **1**. The above described observations suggested that at 340–380 °C inter-blade fusion between neighboring propeller blades of the intermediate compound **5** was initiated. The formation of a single C–C bond between peripheral benzene rings of neighboring propeller blades resulted in a partial planarization, closure of conjoint bay regions and disappearance of the bright lobe in STM images. The presence of molecules exhibiting two (Fig. 3d), one (Fig. 3c) or zero (Fig. 3e) bright lobes in STM topographies might be linked with the formation of one (compound **7**, Fig. 3g), two (compound **6**, Fig. 3f) or three [14]annulene rings (compound **1**, Fig. 3h) within a single molecule, respectively. Such an interpretation seems to be plausible; however, one cannot definitely prove this scenario based solely on STM images of the molecules. In order to dispel doubts we decided to apply nc-AFM imaging with CO functionalized tips allowing for bond resolved analysis of the molecule internal architecture.^12^

**Fig. 4:**
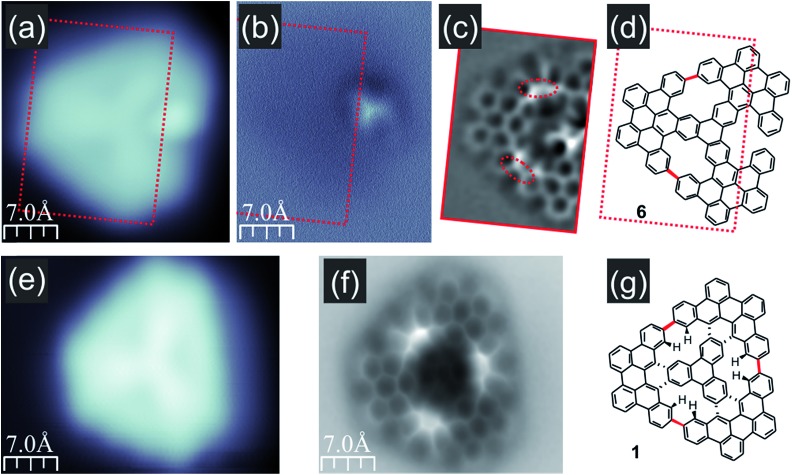
STM and nc-AFM images of compounds **6** and **1**; (a and b) filled state STM and frequency shift nc-AFM images of a molecule with one bright lobe; the AFM image confirms that the bright protrusion in the STM image corresponds to the geometrically elevated part of compound **6**; (c) Laplace-filtered bond-resolved frequency shift nc-AFM image of a flat part of molecule **6** indicated by red rectangles in (a and b) demonstrating the formation of two new C–C bonds (red dashed ovals) between neighboring propeller blades; (d) structure of compound **6** with new bonds highlighted in red; (e and f) filled state STM and Laplace-filtered bond resolved frequency shift nc-AFM images of nanographene **1**; (g) structure of compound **1** with new bonds highlighted in red; imaging conditions: tunnelling current 100 pA, bias voltage –1.0 V.

As the molecules exhibiting three bright lobes (compound **5**) were expected to exhibit a severe non-planar structure, we decided to inspect compound **7** with at greatest two maxima. Imaging at an elevated height confirmed that the bright lobes in STM images came from the significant non-planarity of the molecules (ESI Fig. S4†). We next turned to the molecule with only one bright lobe and anticipated two incorporated [14]annulene units (compound **6**, Fig. 4a and b). The nc-AFM measurement with CO-functionalized tip immediately confirmed the generation of two new C–C bonds between neighboring propeller blades, which led to the closure of two conjoint bay regions of necking and the formation of two fourteen-membered rings as highlighted in red in Fig. 4c and d. The nc-AFM image exhibited a much brighter appearance of the newly created bonds thus revealing the geometrical conformation of the molecule on the surface. It turned out that the central part of the molecule was located much closer to the surface area, while the carbon atoms located at the outskirts of the nanographene flake – which form the new bonds and, consequently, the [14]annulene rings – were raised above the central ring plane. This resulted from the steric hindrance between the hydrogen atoms pointing toward the center of the fourteen-membered ring pore, in agreement with a recent report for the smaller porous nanographene C78 (Fig. 1).^8^ Similar observations could also be made for the target nanographene **1**, which did not exhibit any bright lobes in the STM image (Fig. 4e). The nc-AFM frequency shift image shown in Fig. 4f finally proved the generation of the three-fold symmetric non-planar porous nanographene **1** with three [14]annulene rings embedded (Fig. 4g). Remarkably, the central triphenylene core of the molecule appeared closer to the surface compared to the rest of the molecule.

**Fig. 5:**
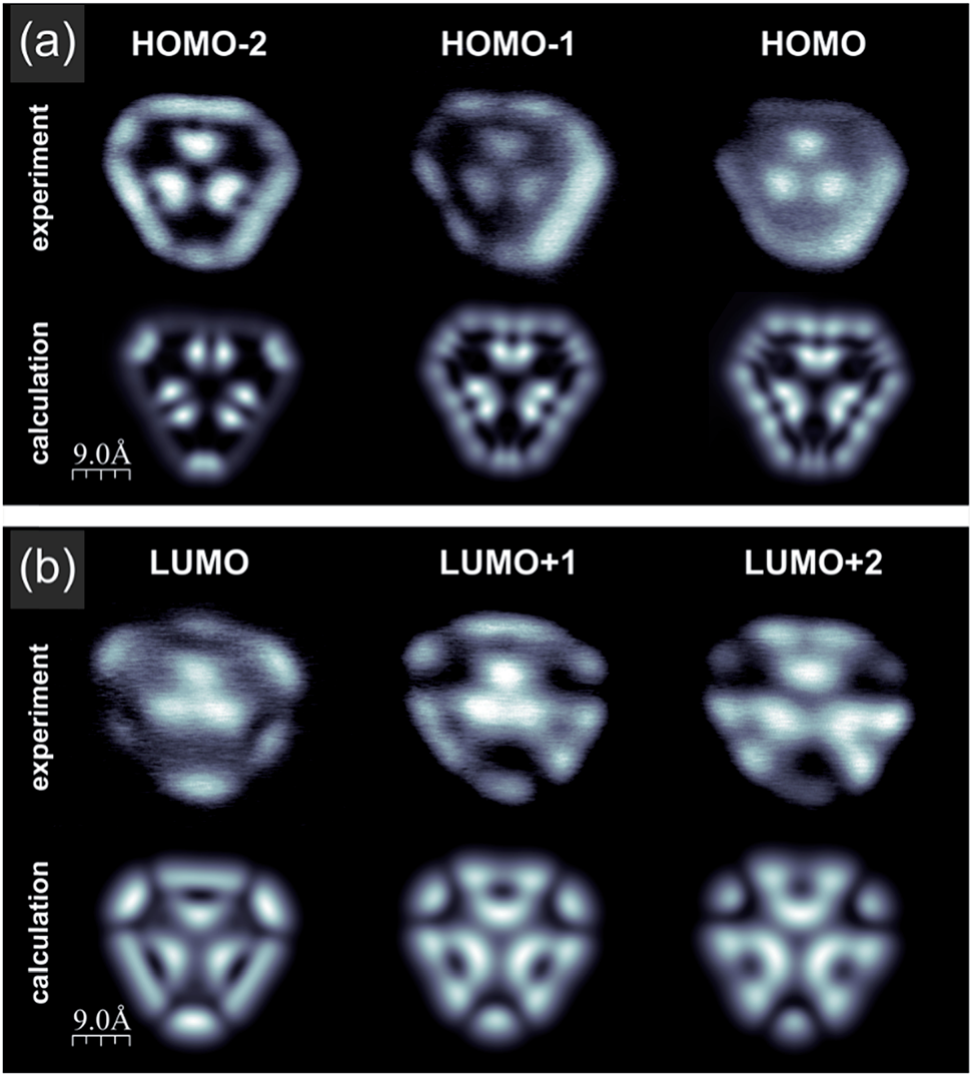
Calculated and experimental constant current d*I*/d*V* images of compound **1** acquired at voltages corresponding to resonances in single point STS data (see the ESI, Fig. S5 and S7†); (a) calculated (upper panel) and experimental (lower panel) filled state d*I*/d*V* maps corresponding to HOMO-2, HOMO-1 and HOMO states; (b) calculated (upper panel) and experimental (lower panel) filled state d*I*/d*V* maps corresponding to LUMO, LUMO+1 and LUMO+2 states; for molecular orbital analysis please refer to the ESI, Fig. S5 and S7.†

Having confirmed the synthesis of porous nanographene **1**, we analysed its electronic structure by means of scanning tunneling spectroscopy (STS). Fig. 5 shows spatial maps of electron clouds acquired at voltages corresponding to resonances registered in single point d*I*/d*V* curves (see the ESI, Fig. S5†). These images are plotted together with the simulated constant current d*I*/d*V* maps exhibiting reasonable conformity. Slight deviations may be attributed to the details of the molecular geometrical conformation and the microscope tip apex structure. Mixing of different molecular orbitals especially for deeper lying states may also play a role.^13^ Due to the large size of the nanographene, full computational analysis of its conformation upon adsorption on Au(111) would require very costly calculations. Moreover, the molecules are expected to interact weakly with the substrate, as demonstrated by the negligible orbital hybridization previously reported for other hydrocarbons.^10^,^14^ Consequently, we decided to perform first principles calculations without the explicit incorporation of the surface, as described in detail in the ESI (Fig. S7†). The overall good consistency of calculated and experimental d*I*/d*V* maps confirms the validity of the applied approximation.

**Fig. 6:**
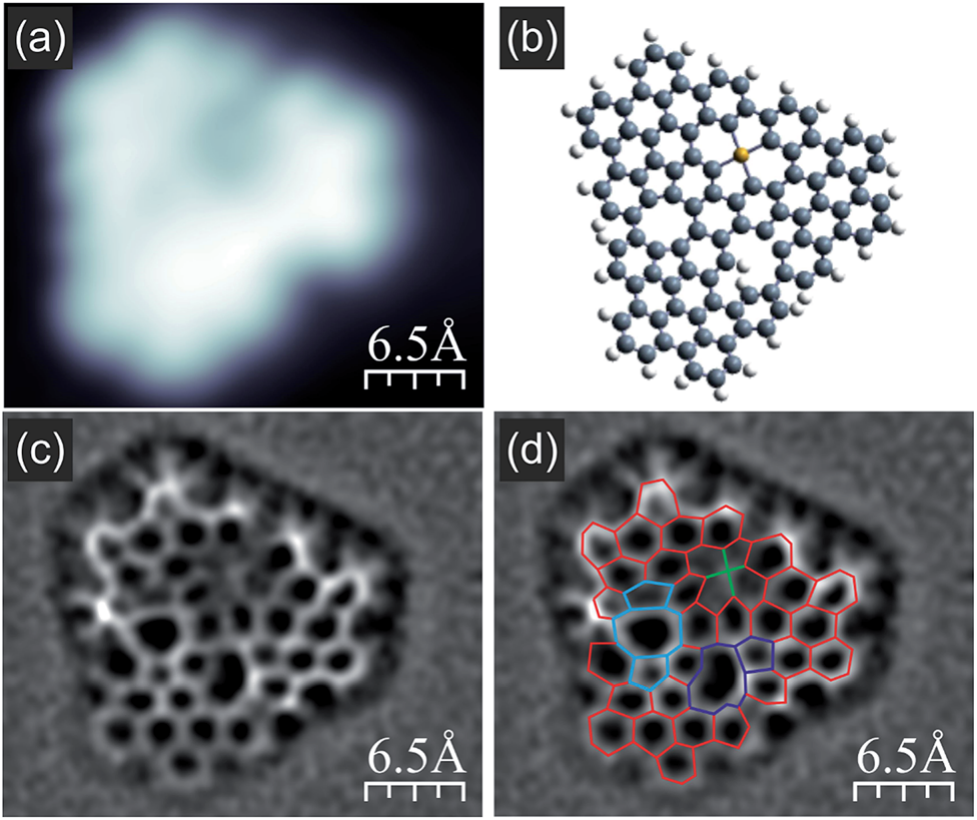
Compound **8**, detected after subjecting nanographene **1** to thermal annealing at 415 °C; (a) high resolution filled state STM topography with two clearly visible slight depressions and one darker depression; (b) anticipated structural (gas phase relaxed) model of the molecule shown in (a) exhibiting reduced symmetry compared to molecules heated up to 370 °C (for details see main text and Fig. S8†); (c and d) Laplace-filtered bond-resolved frequency shift nc-AFM images of the molecule shown in (a), and in panel (d) differences with the molecule containing three [14]annulene units are highlighted; red lines indicate non-modified C–C bonds; blue lines show two [14]annulene pores transformed into the two following units: one containing two pentagonal rings and one octagonal, the second unit built from an undecagonal ring with a pentagonal one attached; green lines indicate the anticipated bonds between the carbon atoms from the third [14]annulene ring and the Au adatom located in the center; imaging parameters: tunnelling current 50 pA, bias voltage –1.0 V.

Interestingly, further increase of the annealing temperature could be used for initiation of new transformations within the [14]annulene pores of nanographene **1**, as shown in Fig. 6. The filled state STM image clearly shows lowering of the image symmetry and the appearance of a dark shadow close to one of the edges (Fig. 6a). The corresponding bond-resolved nc-AFM image (Fig. 6c) allows the unravelling of the new structure (compound **8**, Fig. 6b and ESI Fig. S8†). A closer look indicated that the molecule became completely asymmetric since the three pores evolved differently. In particular, one of the 14-membered rings underwent two cyclodehydrogenations and transforms into a moiety containing two pentagonal rings fused to an eight-membered ring (marked in blue in Fig. 6d). A second [14]annulene underwent only one cyclodehydrogenation resulting in the formation of a pentagonal ring fused to an eleven-membered ring (highlighted in purple in Fig. 6d). The transformation analysis of the third [14]annulene ring led to even more surprising conclusions. This is because the nc-AFM image of this part still exhibited a symmetric appearance, however, with a bright protrusion located in the middle and four noticeable elongated features connecting the central lobe with the surrounding carbon atoms. Such an appearance resembled the presence of an additional atom attached to four C atoms from the 14-membered ring pore. We interpret this additional feature as the manifestation of an Au adatom capture within the pore similar to the self-metalation of porphyrin molecules,^15^ which depending on the oxidation state might exhibit a depression around the additional atom.^16^ The square planar molecular geometry of this cycloaurated structure suggests the formation of an Au(iii) complex with a formal negative charge on the gold atom (ESI Fig. S8†).^17^ Gas phase calculations of compound **8** confirmed the stability of the proposed structure (Fig. 6b), hence predicting significant distortion from the planar conformation mainly due to the steric interactions within the 11-membered ring. The recorded nc-AFM image indicated a less distorted geometrical configuration, which may originate from the influence of the underlying substrate.

## Conclusions

In conclusion, by combining solution chemistry with sequential on-surface reactions, we have synthesized a non-planar three-fold porous nanographene with [14]annulene units embedded. The structure of the target nanographene and the corresponding intermediates has been analysed in detail by means of combined nc-AFM and STM experiments, and the porous nanographene electronic structure has been visualized by STM/STS measurements corroborated by theoretical modelling. The specifically designed precursor, consisting of three centrally fused branches with twelve peripheral phenyl rings, allowed on-surface intra-branch cyclodehydrogenation leading to the formation of a three-blade propeller. The following second-step cyclodehydrogenation processes finally led to the porous nanographene by closing fourteen-membered rings. Overall, twelve new C–C bonds accompanied by nine benzene rings and three [14]annulene pores were generated. By this we set a new path for the synthesis of large exotic nanographenes, where a single-step cyclodehydrogenation is not sufficient for their formation. Finally, we have explored the reactivity of these 14-membered ring pores on a gold surface by visualizing three different transformations within the same nanographene.

## Conflicts of interest

There are no conflicts to declare.

## Supplementary Material

Supplementary information
