# Supplementary material for: Synthesis and reactivity of a trigonal porous nanographene on a gold surface
Source: Chem Sci. 2019 Sep 13;10(43):10143–8. doi: 10.1039/c9sc03404h (PMC6979371; doi:10.1039/c9sc03404h)
Supplement: Supplementary file 1 [file SC-010-C9SC03404H-s001.pdf]

Electronic Supplementary Material (ESI) for Chemical Science.
This journal is © The Royal Society of Chemistry 2019

Electronic Supplementary Information

Synthesis and reactivity of a trigonal porous nanographene on a gold surface

Rafal Zuzak,[a] Iago Pozo,[b] Mads Engelund,[c] Aran Garcia-Lekue,[d] Manuel Vilas-Varela,[b] José M. Alonso,[b] Marek Szymonski,[a] Enrique Guitián,[b] Dolores Pérez,[b] Szymon Godlewski,[a]* Diego Peña[b]*

[a] Centre for Nanometer-Scale Science and Advanced Materials, NANOSAM, Faculty of Physics, Astronomy and Applied Computer Science, Jagiellonian University, Łojasiewicza 11, PL 30-348 Kraków, Poland.

[b] Centro de Investigación en Química Biolóxica e Materiais Moleculares (CiQUS) and Departamento de Química Orgánica, Universidade de Santiago de Compostela, 15782-Santiago de Compostela, Spain.

[c] Espeem S.A.R.L., L-4365 Esch-sur-Alzette,Luxembourg.

[d] Donostia International Physics Center, DIPC, Paseo Manuel de Lardizabal 4, E-20018 Donostia-San Sebastián, Spain & IKERBASQUE, Basque Foundation for Science, E-48013 Bilbao, Spain.

SUPPORTING INFORMATION

Table of Contents — ***Page***

1. Experimental details and spectroscopic data — **S2**

2. Additional nc-AFM images — **S7**

3. Additional scanning tunneling spectroscopy (STS) data — **S8**

4. STM images after 415 °C annealing — **S9**

5. Calculation details — **S10**

6. Structure of compound 8 — **S12**

7. References — **S13**

1. Experimental details and spectroscopic data

1.1. General methods

All reactions were carried out under argon using oven-dried glassware. THF, CH_2Cl_2, CH_3CN and DMF were purified by a MBraun SPS-800 Solvent Purification System. Finely powdered CsF was dried under vacuum at 100 °C, cooled under argon and stored in a glove-box. $Pd(PPh_3)_4$ was prepared from $PdCl_2$ following a published procedure.[1] Other commercial reagents were purchased from ABCR GmbH, Sigma-Aldrich, Panreac or Fisher and were used without further purification. TLC was performed on Merck silica gel 60 F_{254} and chromatograms were visualized with UV light (254 and 360 nm). Column chromatography was performed on Merck silica gel 60 (ASTM 230-400 mesh). ^1H and ^{13}C NMR spectra were recorded at 300 and 75 MHz (Varian Mercury-300 instrument) or 500 and 125 MHz (Varian Inova 500) respectively. MALDI-TOF spectra were determined on a Bruker Autoflex instrument. Melting points were measured by a Büchi melting point B-450 instrument. UV-Vis spectra were measured by a Jasco V-630 spectrometer. Fluorescence in solution was measured in a Fluoromax-2 spectrofluorimeter.

Triflate **3** was obtained in one step from commercially available starting materials, following a published procedure (Scheme S1).[2]

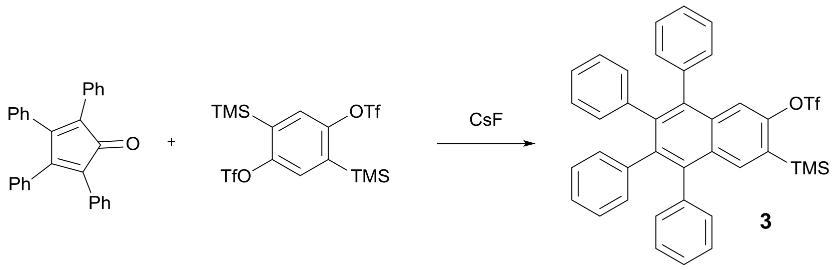

*Scheme S1. Synthesis of triflate **3**.*

1.2. Synthesis of nanographene 2

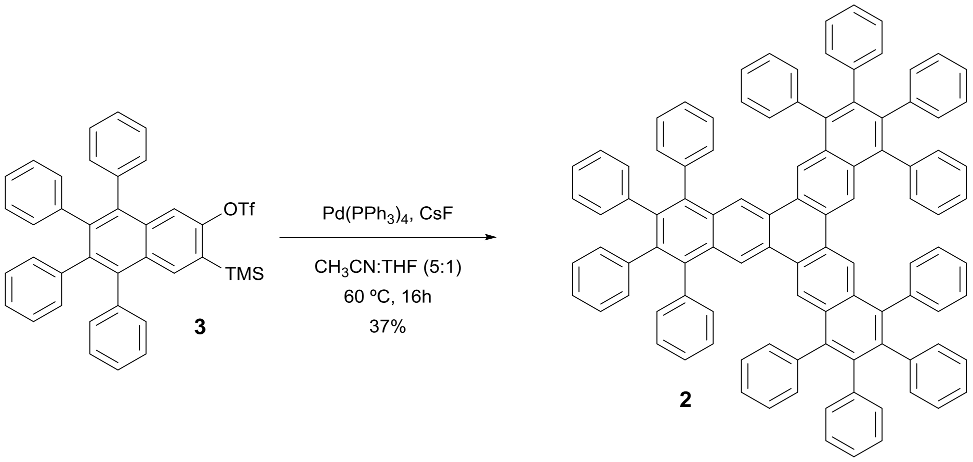

*Scheme S2. Synthesis of nanographene **2**.*

Over a solution of the polycyclic aryne precursor **3** (95 mg, 1 equiv) and $Pd(PPh_3)_4$ (16.5 mg, 0.1 equiv) in CH_3CN/THF (5:1, 12 mL), CsF (65 mg, 4 equiv.) was added. The mixture was heated at 60 °C and stirred for 16h. Then, the reaction crude was filtered and washed with CH_3CN (2x10mL) and Et_2O (2x10mL). The resulting solid was extracted in a Soxhlet apparatus with $CHCl_3$ overnight. The solvent was removed under reduced pressure obtaining product **2** (23 mg, 37 % yield) as a white solid (m.p.: >400 ˚C).

^{1}H NMR (500 MHz, $C_2D_2Cl_4$), δ: 8.42 (s, 6H); 7.21-7.02 (m, 30H); 6.89-6.71 (m, 30H) ppm.

^{13}C NMR-DEPT (125 MHz, $C_2D_2Cl_4$), δ: 131.4 (C), 131.2 (CH), 131.0 (CH), 129.0 (C), 127.4 (CH) 126.9 (CH) 126.4 (CH), 125.1 (CH), 122.1 (CH) ppm.

MS (APCI), m/z: 1291.52 (M^++1).

HRMS (APCI), m/z: found 1291.5235 (calc. for $C_{102}H_{67}$, calcd: 1291.5237).

1.3. Synthesis of nanographene 5

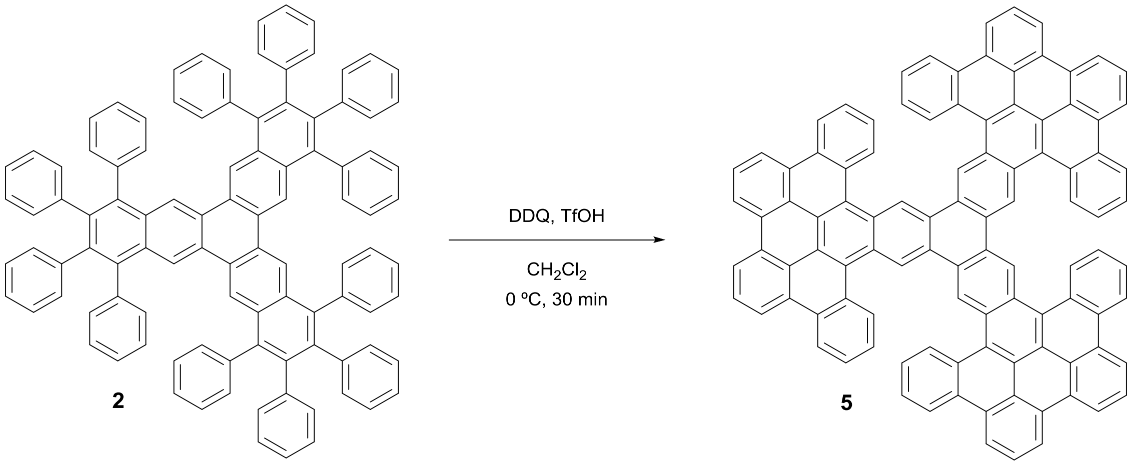

Scheme S3. Synthesis of nanographene ***5****.*

Over a suspension of compound **2** (13 mg, 1 equiv) in CH_2Cl_2 (20mL) at 0 °C, DDQ (23 mg, 10 equiv) was added. After 5 min, TfOH (1 mL) was added and the reaction mixture was stirred at 0 °C for 30 min. Then, 20 mL of $NaHCO_3$ (saturated aqueous solution) were added. The mixture was extracted with CH_2Cl_2 (3x20 mL) and hot $C_2H_2Cl_4$ (5 mL). The volatiles were removed under reduced pressure. The obtained residue was washed with H_2O (2x10 mL), MeOH (2x10 mL) and Et_2O (2x10 mL) obtaining product **5** as a dark solid (3.1 mg). The extreme insolubility of compound **5** precluded its NMR characterization.

MALDI-TOF: 1273.2.

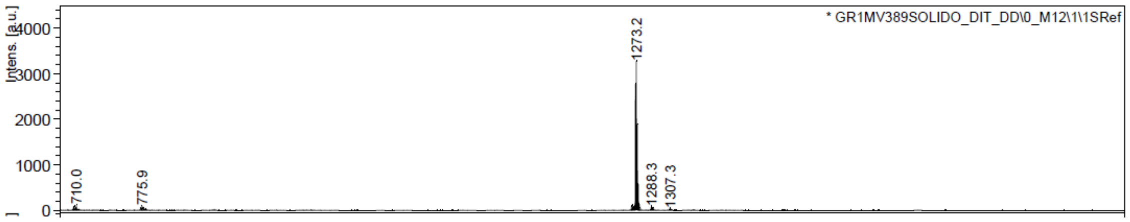

1.4. ^{1}H and ^{13}C NMR spectra of compound 2.

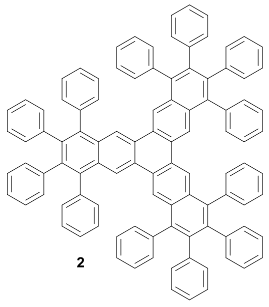

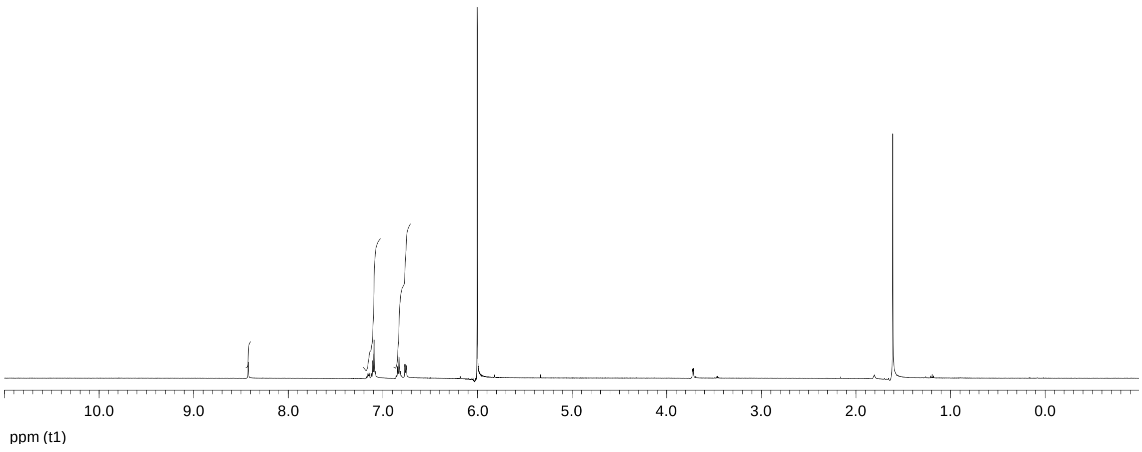

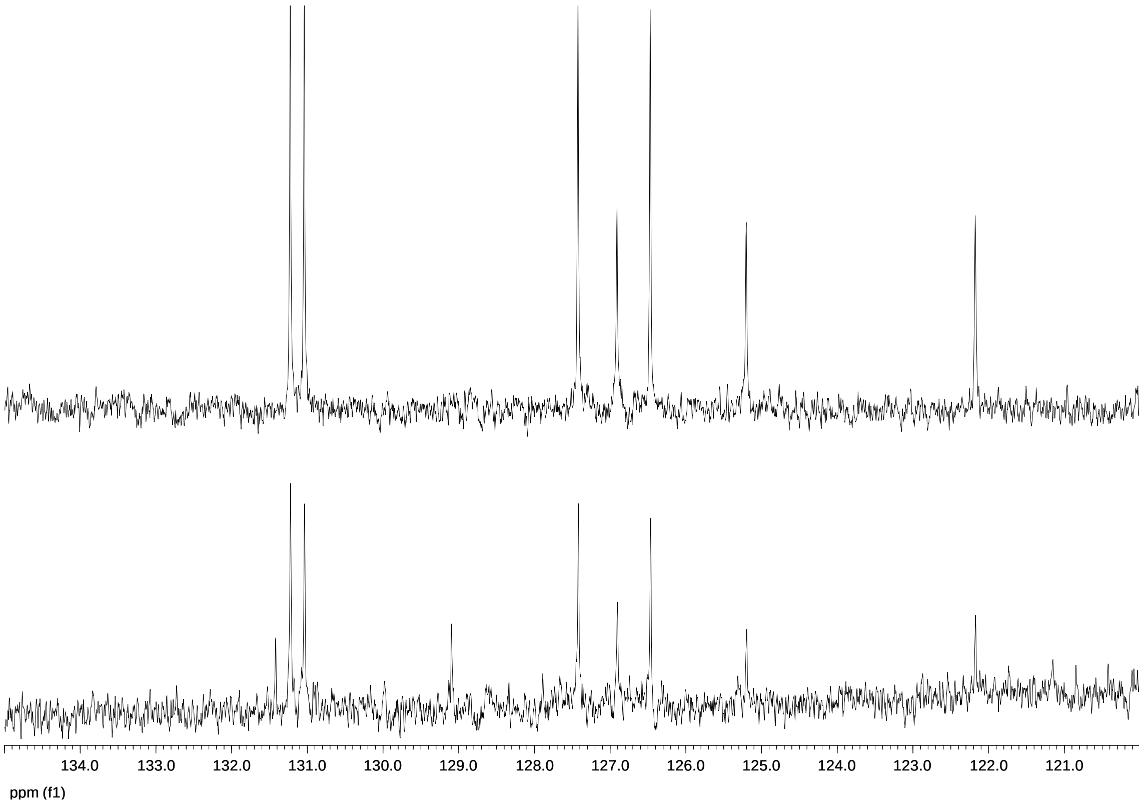

*Figure S1. NMR spectra of compound **2*** in $C_2D_2Cl_4$.

1.5. UV/Vis and emission spectra of compound 2.

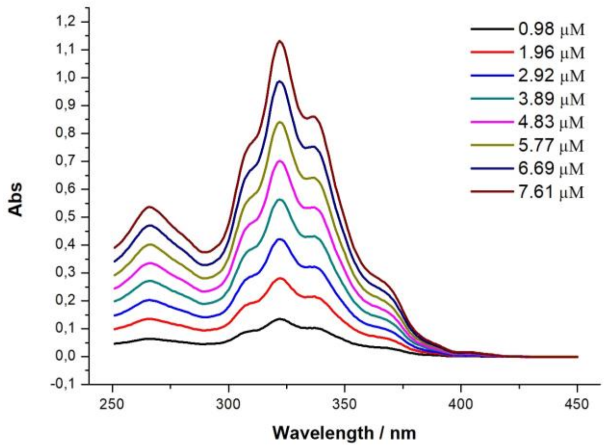

*Figure S2. Absorption spectra of compound **2** in CH_2Cl_2.*

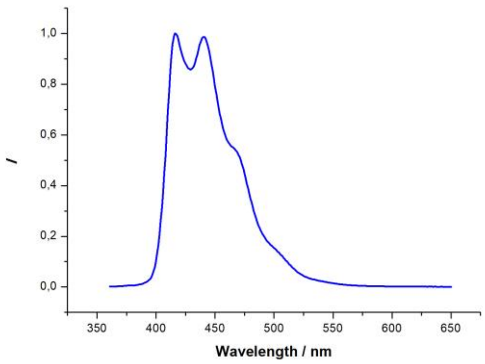

*Figure S3. Emission spectra of compound **2** in CH_2Cl_2.*

.

2. Additional nc-AFM images

Figure S4 shows filled state STM (a) and nc-AFM images (b) of compound **7**, exhibiting only two bright lobe STM contrast. The frequency shift nc-AFM image confirm that the bright lobes recorded in STM correspond to parts of the molecule with increased altitude located within the necking of the conjoined cove regions between the two pairs of neighbouring propeller blades.

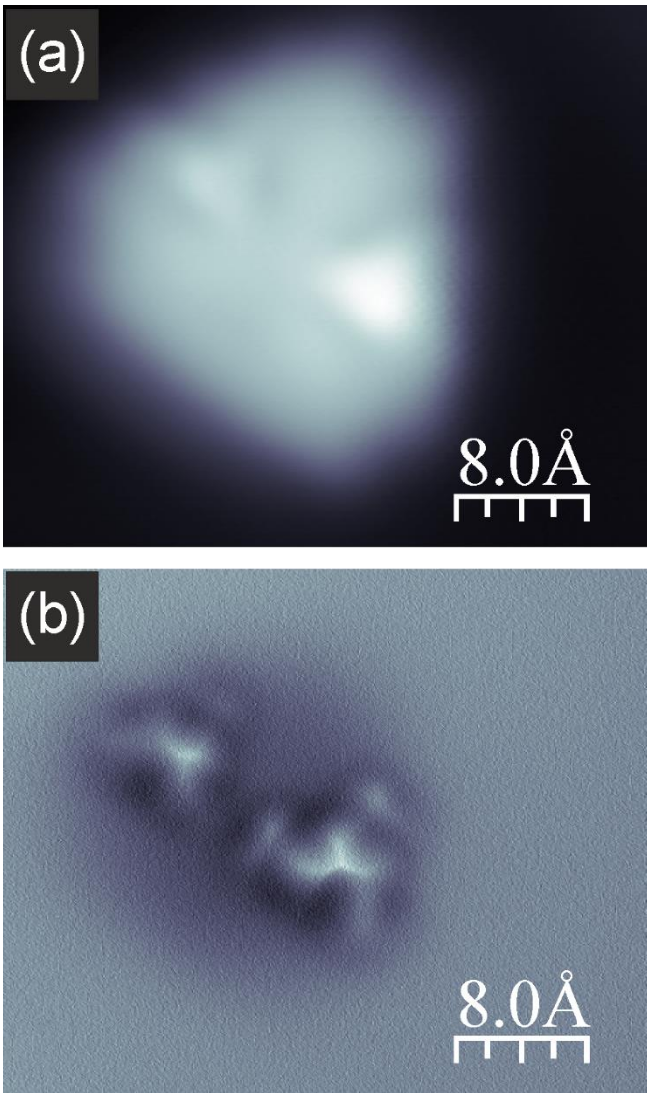

*Figure S4. (a) Filled state STM and (b) nc-AFM images of compound **7** with one [14]annulene pore.*

3. Additional scanning tunneling spectroscopy (STS) data

Figure S5 shows single point scanning tunnelling spectroscopy curves acquired for compound **1**, that is, a molecule with three [14]annulene rings embedded, after cyclodehydrogenation induced at 370 °C. Middle panel shows constant current dI/dV maps recorded for voltages at which single point STS resonances in panels (a, filled states) and (b, empty states) are captured. Comparison between experimental maps and simulated ones is shown for HOMO-2, HOMO-1, HOMO, LUMO, LUMO+1 and LUMO+2 states in Figure 5 of the main text. Interestingly within the filled state part of the STS spectra pronounced resonances in the range of a Au(111) surface state appear. However the spatial dI/dV maps recorded for these voltages do not show any intramolecular contrast over the molecule and we interpret the presence of above described resonances as a manifest of the Au(111) surface state.

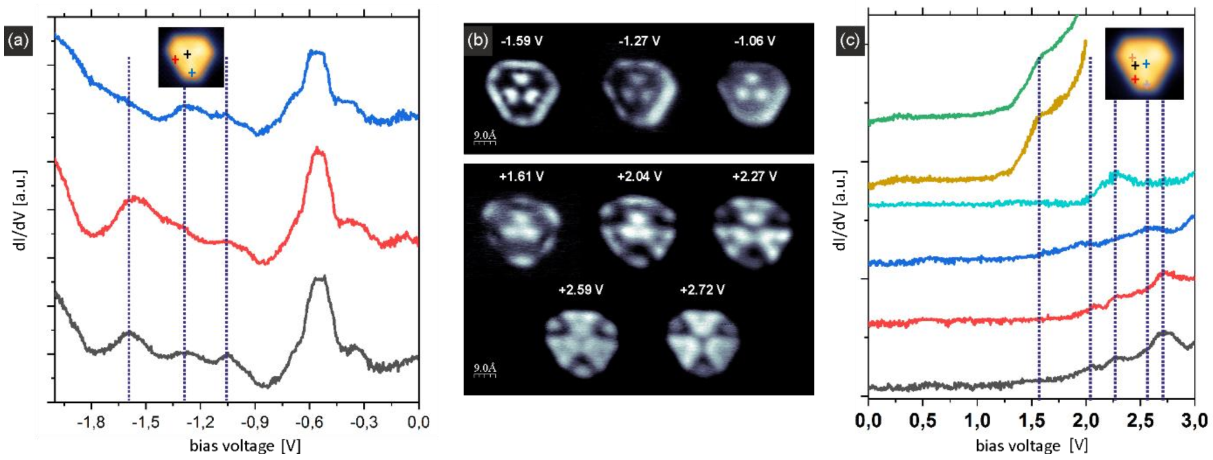

*Figure S5. Scanning tunnelling spectroscopy (STS) data recorded for compound **1** with three [14]annulene rings incorporated; (a) and (c) filled- and empty-state single point STS curves recorded for different planar tip positions indicated by coloured crosses within the insets, (b) spatial dI/dV maps recorded for voltages indicated in (a,c) by vertical dashed violet lines.*

4. STM images after 415 °C annealing

Figure S6 shows additional STM images of the molecules after annealing at 415 °C. Figure S6a indicates that the molecules tend to locate within the elbows of the surface herringbone pattern. Additional high resolution images acquired with the tip functionalized with a CO molecule are shown in Figures S6b,c. In each case the molecular topography contains one darker shadow with a faint lobe in the middle, as well as additional subtle shadows which vary slightly from molecule to molecule. This might indicate on differences in the molecule transformations upon annealing at 415 °C.

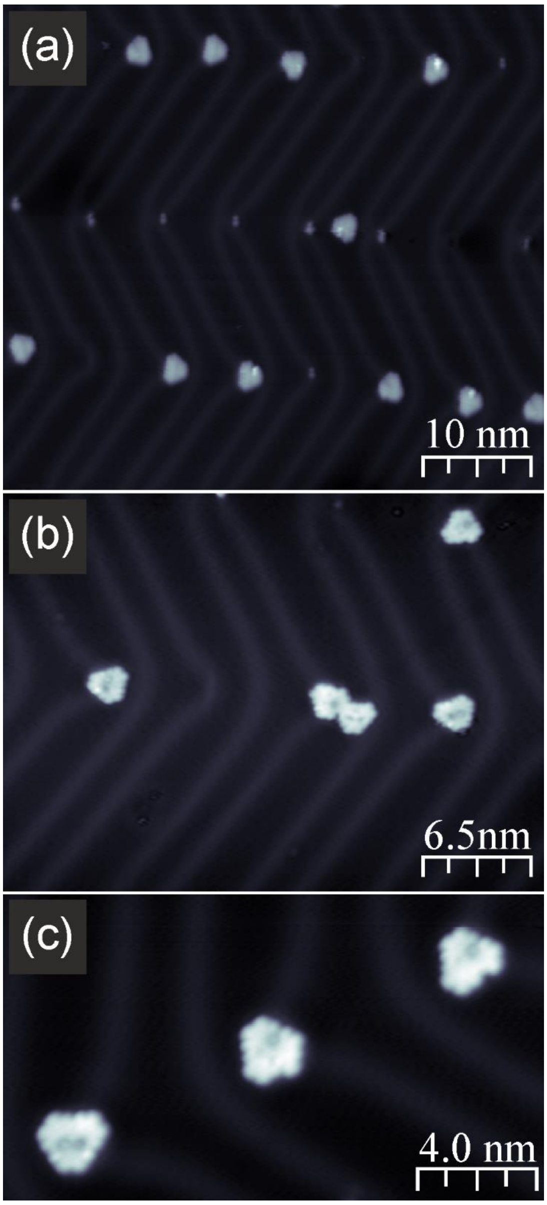

Figure S6. Additional filled state STM images of molecules after 415 °C annealing, (a) overview image indicating on the location of molecules within the elbows of a surface herringbone pattern; (b) and (c) high resolution filled state STM images acquired with the tip functionalized by a CO molecule showing subtle differences between individual molecules, scanning parameters: tunnelling current 50 pA (a,b), 100 pA (c), bias voltage -1.0V

5. Calculation details

Due to the large size of the molecule a full simulation including the Au substrate prove infeasible due to computational cost. In our case, however, the molecule-only model is reasonable, due to the anticipated weak substrate interaction as well as the substantial internal strain in the molecule. However, to retain the alignment effect of a surface we applied a weak asymmetric model potential $P(r) = \frac{a}{r^2} - \frac{b}{r}$ (a=0.2 eV·Å^2, b=0.2 eV·Å).

Geometric relaxations were performed via density-functional theory using the SIESTA code.[3,] Our model: We used a double-zeta-polarized(DZP) basis set with orbital radii defined using a 100 meV energy shift, the Perdew–Burke–Ernzerhof(PBE) version of the generalized gradient approximation for exchange–correlation,[4] a real-space grid equivalent to a 200 Ry plane-wave cutoff. Forces were relaxed until forces were smaller than 0.02 eV/Å.

To compute the STM images we followed the surface integration technique of Paz and Soler. [5] In addition, we used the Tersoff–Hamann approximation [6] assuming a proportionality factor of 1 nA·Å^{-3} for the ratio between the local density of states. dI/dV maps were simulated by first calculating current and differential current independently on a 3D grid and then interpolating the differential current to the current isosurface corresponding to the target current.

In order to mimic the effect of spatial uncertainty in the measurement which reduces the resolution of the STM images, we have convoluted our currents with a Gaussian kernel $K(r, r_0) = (\pi \cdot \sigma^2)^{3/2} \cdot exp\left(\frac{|r-r_0|^2}{2s^2}\right)$. To emulate the experimental process of taking dI/dV maps the tip height was determined using a target current of 1 nA with a, by design, large spatial uncertainty (sigma=2.0 Å), while the differential current was subsequently calculated at a low spatial uncertainty (sigma=0.5 Å). Based on experimental peak widths we used a spectral broadening of 0.2 V.

Molecular orbitals were, as well, generated with the Paz/Soler technique. The isosurface of the absolute value of the orbital was first generated using the density isovalue corresponding to the STM Tersoff-Hamann current isovalue. Then the opposite phases of the orbital were plotted in blue/red while the colour intensity shows the relative spatial height of the orbital (white->black, minimum->maximum) and plotted to give the maximal correspondence with the STM simulations.

For clarity the single electron molecular orbitals are denoted with ‘, whereas molecular states which may be composed of one or more molecular orbitals are denoted without ‘.

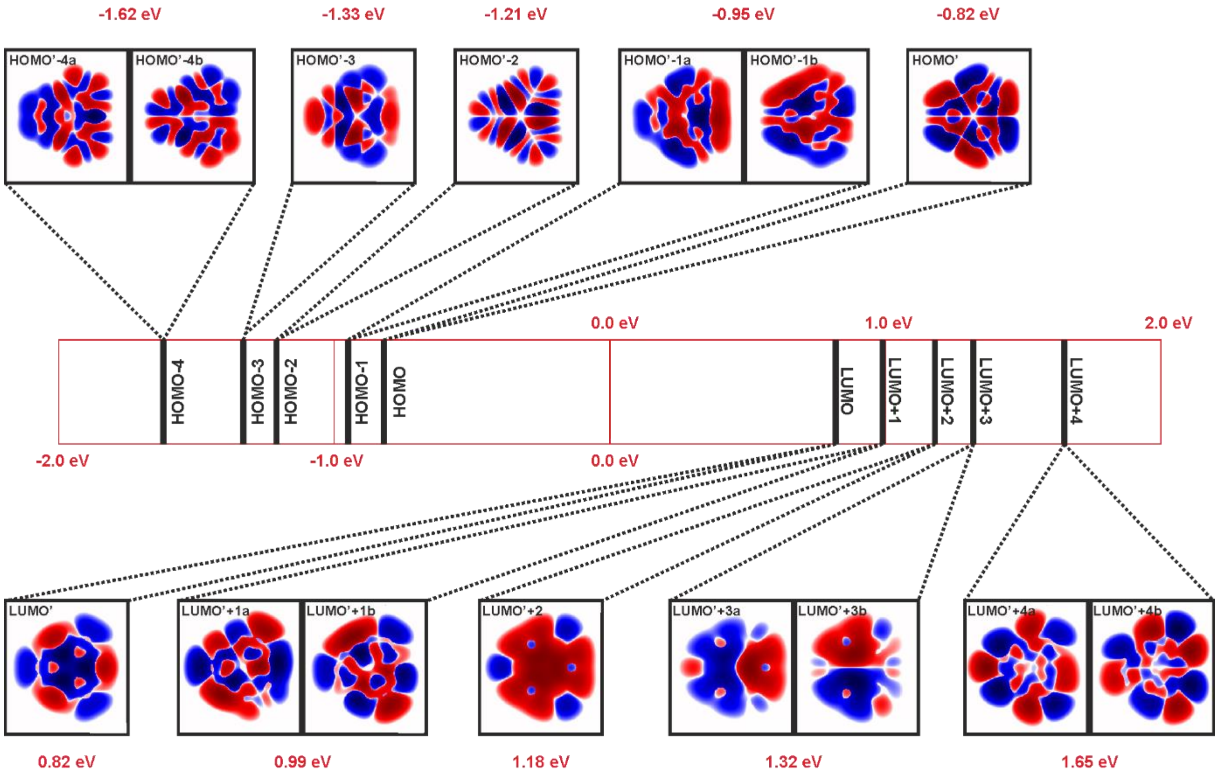

Figure S7. Calculated molecular orbital wavefunctions for compound ***1****, upper panel shows unoccupied molecular orbitals, lower panels presents filled orbitals, middle section indicates the energies of individual molecular states. The middle of the HOMO-LUMO gap is chosen as the zero of energy.*

The geometry of the transformed porous nanographene (Fig. 6b) was optimized using density functional theory as implemented in the SIESTA code.[3] All the atoms were relaxed until forces were < 0.01eV/Ang, and the dispersion interactions were taken into account by the non-local optB88-vdW functional.[7] The basis set consisted of double zeta plus polarization (DZP) orbitals and diffuse 3s and 3p orbitals for C atoms, and DZP orbitals for Au and H atoms. A cutoff of 300 Ry was used for the real-space grid integrations and the Γ -point approximation for sampling the three-dimensional Brillouin zone.

6. Structure of compound 8

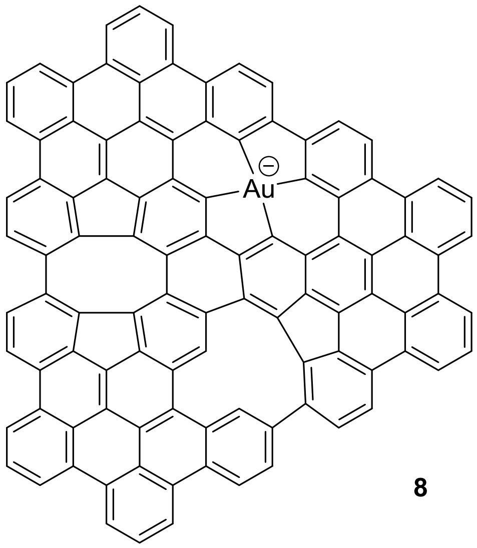

8

Figure S8. Compound **8** detected after subjecting nanographene **1** to thermal annealing at 415 ^{o}C.

7. References

1. (a) L. S. Hegedus, Palladium in Organic Synthesis. In Organometallics in Synthesis: A Manual; M. Schlosser, Ed.; John Wiley & Sons: New York, 1994; (b) M. M. Huq, M. R. Rahman, M. Naher, M. M. R. Khan, M. K. Masud, G. M. G. Hossain, N. Zhu, Y. H. Lo, M. Younus, W. Wong, *J. Inorg. Organomet. Polym.*, 2016, **26**, 1243-1252.

2. D. Rodríguez-Lojo, D. Peña, D. Pérez, E. Guitián, *Synlett*, 2015, **26**, 1633-1637.

3. J. M. Soler, E. Artacho, J. D. Gale, A. Garcia, J. Junquera, P. Ordejón, and D. Sanchez-Portal, *J. Phys. Condens. Matter*, 2002, **14**, 2745-2779.

4. J. P. Perdew, K. Burke and M. Erzerhof, *Phys. Rev. Lett.*, 1996, **77**, 3865-3868.

5. O. Paz and J. M. Soler, *Phys. Stat. Sol. (b)*, 2006, **243**, 1080-1094.

6. J. Tersoff and D. R. Hamann, *Phys. Rev. Lett.*, 1983, **50**, 1998-2001.

7. J. Klimes, D. R. Bowler, and A. Michaelides, *J. Phys. Condens. Matter*, 2010, **22**, 022201.
